# Surgery school—who, what, when, and how: results of a national survey of multidisciplinary teams delivering group preoperative education

**DOI:** 10.1186/s13741-021-00188-2

**Published:** 2021-06-15

**Authors:** I. Fecher-Jones, C. Grimmett, F. J. Carter, D. H. Conway, D. Z. H. Levett, J. A. Moore

**Affiliations:** 1 University Hospital Southampton NHS Foundation Trust, Southampton, SO16 6YD UK; 2 School of Health Sciences, University of Southampton, Southampton, UK; 3 Enhanced Recovery After Surgery (ERAS) UK, 4 Aldon House, Yeovil, UK; 4 Manchester University NHS Foundation Trust, Manchester, UK

**Keywords:** Preoperative education, Surgery school, Prehabilitation, Perioperative medicine

## Abstract

**Background:**

Group education is increasing in popularity as a means of preparing patients for surgery. In recent years, these ‘surgery schools’ have evolved from primarily informing patients of what to expect before and after surgery, to providing support and encouragement for patients to ‘prehabilitate’ prior to surgery, through improving physical fitness, nutrition and emotional wellbeing.

**Method:**

A survey aimed at clinicians delivering surgery schools was employed to capture a national overview of activity to establish research and practice priorities in this area. The survey was circulated online via the Enhanced Recovery after Surgery UK Society and the Centre for Perioperative Care mailing lists as well as social media.

**Results:**

There were 80 responses describing 28 active and 4 planned surgery schools across the UK and Ireland. Schools were designed and delivered by multidisciplinary teams, contained broadly similar content and were well attended. Most were funded by the National Health Service. The majority included aspects of prehabilitation most commonly the importance of physical fitness. Seventy five percent of teams collected patient outcome data, but less than half collected data to establish the clinical effectiveness of the school. Few describe explicit inclusion of evidence-based behavior change techniques, but collaboration and partnerships with community teams, gyms and local charities were considered important in supporting patients to make changes in health behaviors prior to surgery.

**Conclusion:**

It is recommended that teams work with patients when designing surgery schools and use evidence-based behavior change frameworks and techniques to inform their content. There is a need for high-quality research studies to determine the clinical effectiveness of this type of education intervention.

**Supplementary Information:**

The online version contains supplementary material available at 10.1186/s13741-021-00188-2.

The average person in the UK will undergo four to six surgeries during their lifetime (Nepogodiev et al., 2019). Patient education prior to surgery is an established part of the surgical pathway, traditionally undertaken within presurgical clinics and focussing on what to expect. It is generally accepted that well-prepared patients are more confident and less anxious about undergoing surgery (Dawson, 2000). Despite established programmes in the USA since the 1970s (Mezzanotte, 1970), preoperative education in a group setting is a relatively new concept in the UK and Ireland with a primary focus on educating patients within Enhanced Recovery after Surgery (ERAS) programmes, particularly within orthopaedics. Reported outcomes have included reduction in length of hospital stay, reduced preoperative anxiety and reduced postoperative pain (Giraudet-Le Quintrec et al., 2003; McGregor et al., 2004).

The last 5 years have seen an evolution of ‘surgery schools’ within the UK and Ireland. The schools now not only prepare patients for what to expect, but also provide advice and support on what patients can do to prepare themselves physically and mentally to reduce the risk of postoperative complications (Moore et al., 2017; Moore et al., 2020). Improving physical fitness, nutrition and emotional wellbeing prior to surgery is known as ‘Prehabilitation’ and has been shown to improve surgical outcomes (Li et al., 2013). To date prehabilitation interventions have primarily been limited to research trials. There is now emerging evidence that educating surgical patients in groups as part of a clinical service and using evidence-based behaviour change techniques may promote behaviour change, thus optimising physical and psychological health (Fecher-Jones et al., 2021).

Despite these ‘Fit for Surgery’ schools anecdotally growing in number and becoming a regular feature of perioperative conference agendas, clinical and patient-reported outcomes of these interventions remain largely unreported. As leads for surgery schools at University Hospital Southampton and Manchester Royal Infirmary, we felt it timely to capture a national overview of surgery school development and activity. The aim was to identify similarities and differences in surgery schools across the UK and Ireland, including content, outcomes measured and funding mechanisms. The results would enable greater insight into the variations of this educational intervention and allow research and practice priorities to be identified.

## Methods

A bespoke survey was conducted in the summer of 2019 aimed at health care teams delivering surgery schools (See Additional File 1).

The survey was designed using ‘Survey Monkey’ (Survey Monkey, 2019), an online survey platform. The link to the survey was initially sent out via email to all members of the ERAS UK society as a pilot to gauge responses, and following minor amendments was disseminated by the authors through social media (Twitter and Facebook) from 13/9/2019. Response rate was reviewed, and the survey sent out again by the UK Centre for Perioperative Care to all perioperative medicine leads. The survey was open for 94 days, closing on 16/12/2019. Descriptive statistics were calculated for numeric survey items. Content analysis was used to analyse open responses and key themes identified.

## Results

**Fig. 1:**
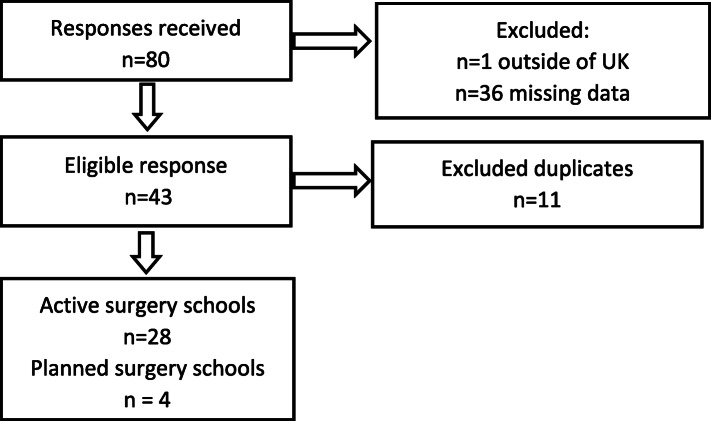
Breakdown of survey responses

There were 80 responses to the survey, from 45 different hospitals. One respondent was excluded as they were from outside of the UK and Ireland and 11 others excluded as they were duplicate entries. Thirty-six respondents had significant missing data, providing job title and location only. Following exclusion of duplicated entries and missing data, there were 28 active surgery schools and four with planned start dates. For breakdown of responses see Fig. 1.

The active and planned schools were situated in 23 different National Health Service (NHS) Trusts with broad spread across England, Scotland and Wales and one in Dublin. There was no reply from colleagues in Northern Ireland. Some geographic areas such as London had more than one surgery school. One respondent who described an active school declined to enter their geographical location.

Over half of the respondents of active and planned schools were anaesthetists 24 (56%), and 12 (28%) nurses. The remaining six respondents were other members of the multidisciplinary team (MDT) including physiotherapists, occupational therapists and one surgeon.

### Surgery school design

**Fig. 2:**
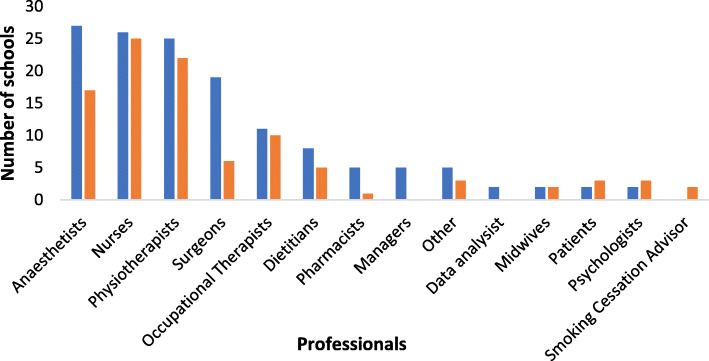
MDT involved in surgery school design (blue) and delivery (orange)

Respondents described a range of MDT members involved in the design and delivery of the 28 active and four planned schools (see Fig. 2). Most common professions involved in design were anaesthetists, nurses, physiotherapists and surgeons, with psychologists and patients in the minority. Over half of the responders (66% (*n*=32)) stated that their school was delivered or would be delivered by three or more members of the MDT. Most commonly, these included a nurse, physiotherapist and anaesthetist.

A small number (11% (*n*=4)) of surgery schools had been running for over a decade, most commonly in orthopaedics. Almost half (43% (*n*=12)) had started within the last 2years and reported finding it useful to visit other hospitals running schools before setting up their own..

Most respondents (71% (*n*=23)) reported that their surgery schools were funded by their own NHS Trust. The remaining schools were funded by local partnerships, national grants and charities; two of the schools were unfunded and undertaken in staff members’ own time. Almost half (46% (*n*=15)) reported that their funding was ongoing, 18% (*n*=6) reported fixed term funding and the remaining 24% (*n*=7) did not know their funding mechanism.

### Surgery school structure and content

The majority of active surgery schools (71% (*n*=20)) were reported as lasting 1 to 2 h and offered as a single session per patient (85% (*n*=24)), with between 5 and 20 patients (82% (*n*=23)) attending each school event. Three respondents stated that they had more than twenty patients attend at a time. Patient attendance was reported by 82% (*n*=23) of respondents and ranged from 35 to 100% of those invited, with the majority (78% (*n*=22)) reporting very good attendance rates of 80–100%.

**Fig. 3:**
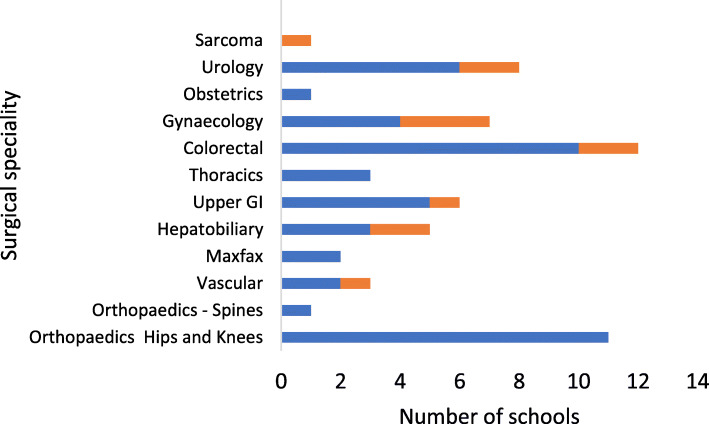
Specialties invited to active surgery schools (blue), specialties to be invited to planned schools (orange)

Active schools were being delivered to ten different surgical specialties, mostly commonly in orthopaedics, colorectal and urology (see Fig. 3). Orthopaedic schools (*n*=12) were run exclusively for orthopaedic patients. The remaining schools apart from three colorectal schools were mixed specialty.

Eleven of the hospital teams (39%) invited all their surgical patients undergoing major surgery to attend their surgery school as part of the surgical booking process, with three of those (11%) stating that school attendance was mandatory for patients prior to surgery. The remaining 61% (*n*=17) of active schools relied on case by case referrals from their surgeons, clinical nurse specialists and preoperative teams.

### Taught content

**Fig. 4:**
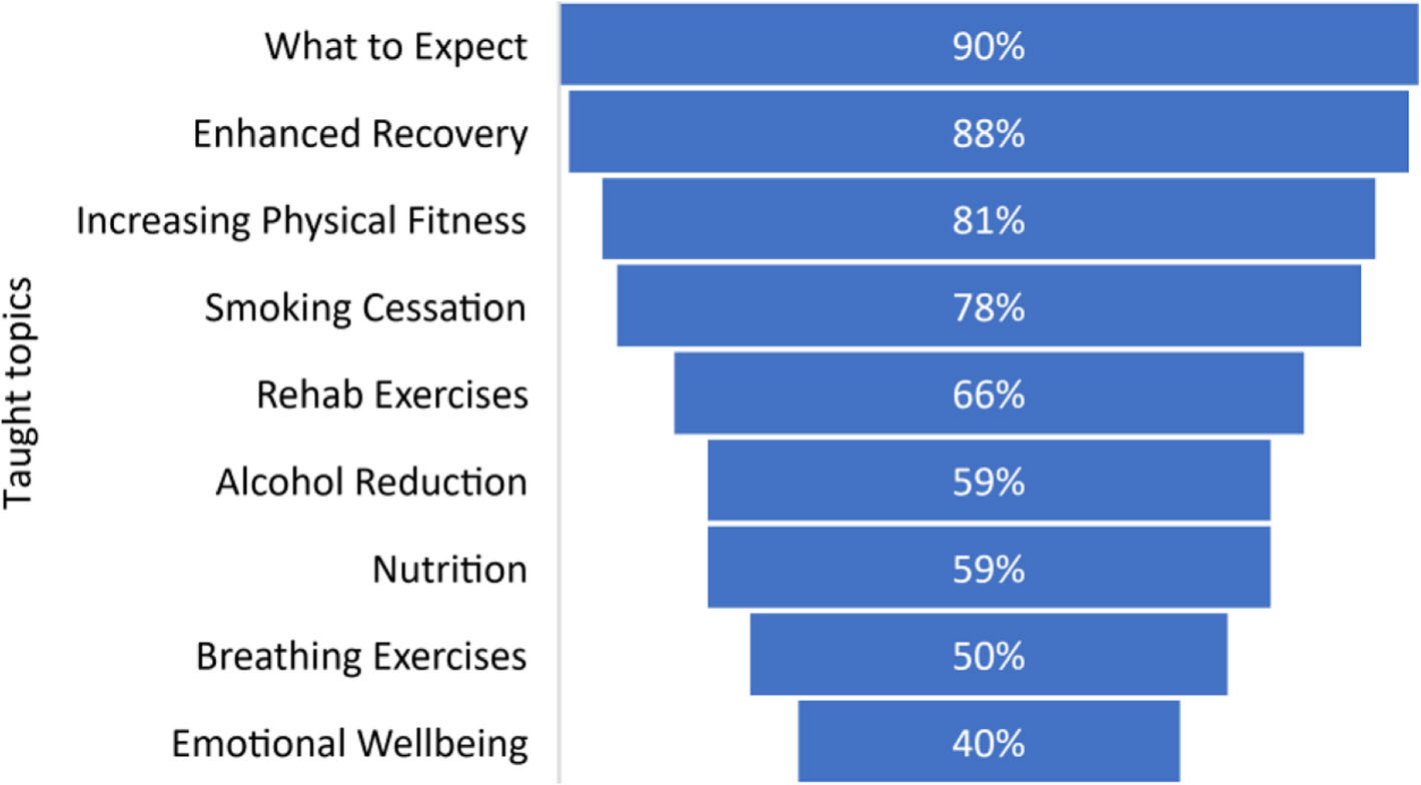
Topics taught at surgery school by popularity

The active and planned schools covered a range of topics. Most commonly taught were what to expect coming into hospital, enhanced recovery principles, increasing physical fitness, rehabilitation exercises and smoking cessation. Only half of the schools taught postoperative breathing exercises, and less than half described specific content to support emotional wellbeing (see Fig. 4). Other topics included stoma care training, pain control and the role of therapy services in recovery.

Almost all respondents (96% (*n*=31)) reported using supporting resources alongside their face-to-face teaching; most commonly used (75% (*n*=24)) were bespoke locally designed patient information leaflets; only 19% (*n*=6) used national written patient information. Almost half of the schools sign-posted to online resources (47% (n=15), videos and DVDS were used by 41%  (n=13)), and less commonly Apps (13% (n=4)), DVDs, apps and signposting to online resources.

The majority of active and planned schools (78% (*n*=25)) used the patient contact opportunity to undertake further screening and assessment including screening for anaemia (28% of schools (*n*=9)) and recruitment for research studies (22% (*n*=7)). Other screening was undertaken for physical fitness (19% (*n*=6)), malnutrition (16% (*n*=5)), anxiety (9% (*n*=3)), lifestyle factors (3% (*n*=1)) and frailty (3% (*n*=1)).

### Behaviour change support

Respondents were asked if they offered behaviour change support as part of their surgery school. Sixty-six percent (*n*=21) described using at least one method of support, including encouraging patients to set goals, keep diaries and use methods of communication during the session to encourage and motivate attendees.

**Fig. 5:**
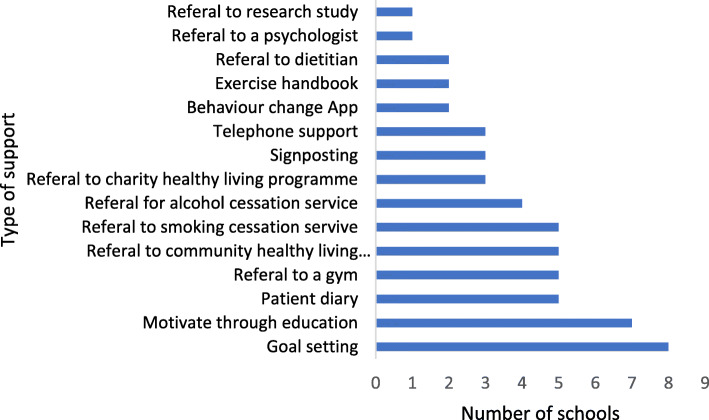
Behaviour change support used in surgery schools

Referral to agencies to support behavior change such as charities, gyms, council programs and smoking and alcohol cessation services was frequently described (see Fig. 5), as well as other NHS services such as dietitians, psychology and access to telephone support with session facilitators.

Just over one third of respondents of active and planned schools (*n*=12) reported developing collaborations with local organisations to support the prehabilitation of patients including local cancer charities (22% (*n*=7)) and local authority programs (16% (*n*=5)), see Table 1.

**Table 1:** Surgery school collaborations

Organisation	Number of schools
Cancer charities	7 (22%)
Local authorities	5 (16%)
Local gyms	2 (6%)
Research studies	2 (6%)
Sports charities	1 (3%)
Universities	1 (3%)

### Outcome data

Patient outcome data following surgery school was collected by 75% (*n*=21) of active schools. Fourteen percent (*n*=4) did not collect any data, and 11% (*n*=3) did not know if they did. Forty-three percent of active schools (*n*=12) recorded length of hospital stay and 39% (*n*=11) postoperative morbidity and mortality. Other outcomes measured were postoperative data on time to drinking, eating and mobilising (25% (*n*=7)); patient satisfaction (21% (*n*=6)); patient-reported outcome measures (14% (*n*=4)); and behaviour change (7% (*n*=2)).

### Future plans

The majority of active schools (75% (*n*=21)) reported ambitions for the future of their surgery school which included expanding to other specialties and hospitals (39% (*n*=11)), developing a complete prehabilitation service (25% (*n*=7)) and developing online support material for patients (25%). Others aimed to improve attendance (18% (*n*=5)), integrate more behaviour change interventions (18% (*n*=5)) and collect more outcome data (7% (*n*=2)).

## Discussion

These novel survey results provide an insightful overview of surgery school activity across the UK and Ireland and confirm growing integration of prehabilitation.

Most respondents were anesthetists or nurses with notably only one surgeon. This may be due to survey distribution bias; although ERAS UK and Centre for Perioperative Care are both multidisciplinary organisations, the membership spread across the professions is not known and preoperative optimisation tends to be anaesthetic led. Respondents reported involvement from a wide range of the MDT in the design and delivery of their schools, predominated by anaesthetists, surgeons, nurses and physiotherapists, which concurs with the published literature regarding the design and delivery of surgery schools (Moore et al., 2017; Fecher-Jones et al., 2021; Shuldham et al., 2002).

Only two (7%) of teams involved patients in the design of their schools and 11% in the delivery. Patient participation in the design, delivery and evaluation of new services has been found to enhance service delivery and acceptability (Bombard et al., 2018). Service users are also key stakeholders in any intervention that aims to change behavior (NICE, 2014). Surgery schools aim to empower patients and their support network to take an active role in planning and preparing for their operation (Royal College of Anaesthetists, 2017); therefore, their role in the planning and design phase of the school is important if it is to achieve its aim.

To our knowledge, there is only one example in the published literature of utilising patient and family input to support the design of surgery schools and other education-based resources (Moore et al., 2017). This concurs with the low number of respondents to the current survey describing such involvement. However, a significant number of respondents reported working with local organizations and explicitly cancer charities, which would help recognise the needs of patients and their families. Considering local patient and family participation in surgery school design and implementation is recommended for future services.

Most of the schools aimed to support patients to increase their physical activity and/or make healthful dietary changes, yet only two reported psychologist involvement, and none recount involvement of a behaviour change specialists in the design of the schools. The design of behaviour change interventions should be evidence-based, patient-focused and delivered by teams with core skills, knowledge and competence in behaviour change techniques (NICE, 2014). Over half of the respondents reported using methods to help motivate and facilitate patients’ behaviour change but were unable to link these with evidenced-based behavior change techniques (Michie et al., 2013). This suggests a lack of knowledge of behavior change models and techniques and supports the case for behavior change specialists to be involved in surgery school design.

One third of the respondents reported working collaboratively with local agencies such as charities and local authorities to support patients to make lifestyle changes. Identification and signposting to support networks is critical if behaviour change is to be achieved (NICE, 2014; Grimmett et al., 2021). It is well evidenced that many people will require more than just instruction to change behaviour. More intensive personalised support may be required in order to make and sustain lifestyle behaviour changes (Grimmett et al., 2019).

The notable increase in the numbers of new schools in the last 2 years reflects the growing interest in prehabilitation particularly for cancer (Macmillan, 2019) and the ability to integrate aspects of this into surgery school curriculums as a universal intervention for all surgical patients. Respondents also reported visiting other surgery schools within the UK which they encouraged. Visiting and talking to other clinicians about their surgery school appears to promote shared learning and prevents teams from ‘reinventing the wheel’. It may however have limited the development of different models of surgery school. Indeed, the schools described in this survey appear very similar, with most lasting 1 to 2 h, organised as a one-off session and attended by 5–20 patients at a time. Surgery schools were generally offered to a wide range of surgical specialties, although notably absent was cardiac surgery, which was unexpected given there are published studies demonstrating the benefit of group education on the clinical outcomes of cardiac patients (Shuldham et al., 2002; Goodman et al., 2008). It is also noted that 40% (*n*=11) of the active schools were Orthopaedic. These ‘Joint schools’ were more likely to be longer established but less likely to include elements of prehabilitation. This is supported by the literature, where joint schools are commonly described as standard care prior to joint surgery, often delivered by nurses and surgeons with more of a focus on rehabilitiation than prehabilitiation (McDonald et al., 2014).

Most schools taught similar topics, with emphasis on enhanced recovery and patient expectation management of major surgery. Prehabilitation components were also common, including increasing physical activity taught by 81% of schools and preoperative nutrition taught by 59% of schools. Support for psychological wellbeing was included in just 40% of schools, despite being a key component of trimodal prehabilitation (Li et al., 2013), and with evidence that poor preoperative psychological health is associated with poorer outcomes (Rosenberger et al., 2006; Kitagawa et al., 2011). However, research in this area is in its infancy, with an urgent need for high-quality trials investigating the impact of preoperative psychological support (Levett & Grimmett, 2019). Although preoperative nutrition was taught by two thirds of the schools, only 25% listed a dietitian as part of their design team, compared with 78% schools who listed physiotherapists. This is despite convincing evidence that preoperative malnutrition is associated with a worse postoperative outcome (Gillis & Wischmeyer, 2019).

The majority of schools supplemented teaching with additional learning resources with most offering bespoke, locally written information. Less than 20% (*n*=6) reported using nationally available patient information such as the Royal College of Anaesthetists ‘Fitter Better sooner’ guide (Royal College of Anaesthetists, 2017), perhaps suggesting that the national documentation does not quite fit local requirements. It has been argued that written documentation is often ineffective in communicating information due to issues with readability and accessibility (Zorn MaR, 2000). Evidence suggests that between 43 and 61% of English working age adults routinely do not understand health information due to low health literacy (Rowlands et al., 2015). In relation to prehabilitation, there is a strong association with health literacy and physical exercise; the higher the health literacy, the higher the frequency of physical exercise. It is also known that patients with lower health literacy have more difficulty in planning and adjusting their lifestyle to maintain good health (Wittink & Oosterhaven, 2018). Web- and phone-based apps have been found to be an effective way of delivering health information tailored to individuals with limited health literacy (Kim & Xie, 2015). Results from our survey found that only three respondents reported using apps, but many mentioned them in their future ambitions.

Although the majority of respondents reported their schools to be NHS funded, only 25% of these charged a tariff (an agreed price for an individual service). Some Trusts may receive ‘block payments’ based on an agreed payment for the provision of a service rather than individual payment per appointment or episode. Funding surgery schools is anecdotally a challenge. We found that some teams were delivering schools in their own unpaid time. Under half of respondents had secured long-term funding. Group education sessions are a cost-effective way of information giving to patients (Seesing et al., 2014) and may reduce the outpatient time needed. Given their potential for reducing surgical complications and length of stay, they may present an attractive cost improvement opportunity for organisations. However, the limited evidence base for the efficacy of surgery schools may provide an explanation as to why organisations are hesitant to commit to funding.

Seventy five percent of respondents reported collecting outcome data; however, postoperative length of stay and morbidity and mortality were collected by fewer than half of the teams, and behaviour change by only 7%. Without these as markers of clinical effectiveness, sustaining the service within the current financial climate is likely to be difficult. Given the additional staffing resources that are often needed to collect robust prospective data, there is a need for standardisation of a minimum dataset, including measures of behavior change, length of hospital stay, post-operative morbidity as well as patient-reported outcome measures. Data collection tools are also needed to facilitate this practice. Including a patient education category within national perioperative audit datasets such as The Perioperative Quality Improvement Project (Health Services Research Centre Perioperative Quality Improvement Project (PQIP), 2019) would be one way of doing this.

This survey was conducted before the COVID-19 pandemic, which resulted in face-to-face group education no longer being an option due to the risk of virus transmission. Five of the centres who took part in this survey have subsequently contacted the authors and report moving their surgery schools to online group sessions in recent months. The findings of this survey therefore remain highly relevant regarding the future of group preoperative education whether it be delivered virtually or face-to-face. The pandemic has also resulted in a backlog of patients awaiting surgery, thus creating an extended window of opportunity for patients to improve their fitness for surgery through lifestyle modification. Surgery school provides a likely cost-effective platform for perioperative clinicians to support patients to use this time proactively which should not be overlooked.

### Strengths and Limitations

This survey provides the first published window of insight into surgery schools across the UK, establishes a baseline of clinical activity and identifies the similarities and differences between schools. Capturing this has enabled us to identify clear suggestions for practice and research that will underpin the future of surgery schools.

Although 80 clinicians responded to the survey only, 32 different schools were identified, which equates to 9% of 356 UK hospitals undertaking surgery having a surgery school (National Audit Project (NAP), 2021). We acknowledge that the actual number of surgery schools in the UK will likely be higher and that there may be bias in responses by nature of the fact that those with an interest surgery schools were more likely to take part. It was also noted that although responses were from across the UK and Ireland, they were most commonly from larger centres within UK cities, and almost a third were from London and the South of England. This bias may be due to those centres having more established perioperative services. The reasons for the large number of responses with missing data are also not known. It would have been useful to include within the survey a question asking whether respondents had a surgery school. A response of ‘no’ would have justified missing data. Responses to the open questions varied considerably in length and depth. This may have been because some respondents had more time or insight into the detail of their surgery school than others, but none the less will have influenced the overall findings.

### Suggestions for practice and research

Future priority is to establish the clinical effectiveness of surgery school. This relies on teams and organisations working together to standardise their data collection and publish their outcomes. Data collection of outcomes needs to be considered within service development plans and adequately resourced. The recently completed Greater Manchester Implementation of ‘ERAS+’ will provide useful information about the effectiveness of surgery school (The Health Foundation, 2020).

Teams aiming to change patient behaviour should involve service users in the design of their programmes as well as evidence-based techniques for supporting behavior change.

Health literacy of participants should be considered from the outset to ensure accessibility of surgery school for all patient participants and the most appropriate supporting resources.

## Conclusion

Surgery schools are a growing phenomenon in the NHS and provide education on a range of topics to patients and their families prior to major elective surgery. Schools provide a platform for introducing the elements of prehabilitation and the potential for motivating behaviour change. Inclusion of behavioral science and patients within the design of these interventions would maximise the effectiveness of schools in promoting behaviour change. Funding remains the biggest threat to the future of these schools, and without comprehensive collection of clinical effectiveness outcome measurements, and dissemination of the results, the future is uncertain. The authors challenge teams to think creatively, particularly around this time of uncertainty within the NHS, to establish collaborations with external agencies and focus on developing and sharing resources to improve access for all patients to this type of education.

## Supplementary Information

**Additional file 1.** Survey questions

## Data Availability

All survey data is available from the corresponding author on reasonable request.

## Abbreviations

ERAS: Enhanced Recovery after Surgery
NHS: National Health Service
MDT: Multidisciplinary team
BCT: Behaviour change technique
