# Supplementary material for: Surgery school—who, what, when, and how: results of a national survey of multidisciplinary teams delivering group preoperative education
Source: Perioper Med (Lond). 2021 Jun 15;10:20. doi: 10.1186/s13741-021-00188-2 (PMC8203307; doi:10.1186/s13741-021-00188-2)
Supplement: Supplementary file 1 — Additional file 1. Survey questions [file 13741_2021_188_MOESM1_ESM.docx]

**Additional File 1** Survey questions

| **Question Number** | **Question** | **Answer Format** |
| --- | --- | --- |
| 1 | What is your job title? | Free text |
| 2 | What is your place of work? | Free text |
| 3 | Which members of the MDT were involved in the design of your school | Free text |
| 4 | When did you start running your school? | Free text |
| 5 | Did you visit any other surgery school? If yes where. | Closed question |
| 6 | How did you get funding to help you start your surgery school? | Tick box options |
| 7 | What is the duration of this funding? | Free text |
| 8 | How long does your school last? | Tick box options |
| 9 | Is it a one of session? If no please describe follow up. | Closed question |
| 10 | How many patients attend each session? | Tick box options |
| 11 | What specialties are included? | Free text |
| 12 | Who refers the patients to you? | Tick box options |
| 13 | What is the attendance rate of those invited? | Free text |
| 14 | Who delivers the school? | Tick box options |
| 15 | What topics are covered? | Tick box & free text |
| 16 | What other supporting materials do you use? | Tick box & free text |
| 17 | Do you use the patient contact opportunity to do other activities? | Tick box & free text |
| 18 | What methods do you use to support patients to make any changes to their behaviour i.e. to increase physical activity? | Free text |
| 19 | Do you use any specific behaviour change techniques to support patients to change their behaviours prior to surgery? For example encouraging goal setting or self-monitoring of behaviour. | Tick box & free text |
| 20 | Do you collect any outcome data following patients’ attendance at school? | Tick box & free text |
| 21 | Do you charge a tariff for patient attendance? | Tick box & free text |
| 22 | Are you working with any local teams to undertake prehab for your patients? | Tick box & free text |
| 23 | What are your future plans for your surgery school? | Free text |
| 24 | What advice would you give to another hospital starting a surgery school? | Free text |
| 25 | If you have any comments or suggestions related to this study? | Free text |
